# CoMI: consensus mutual information for tissue-specific gene signatures

**DOI:** 10.1186/s12859-022-04682-2

**Published:** 2022-04-19

**Authors:** Sing-Han Huang, Yu-Shu Lo, Yong-Chun Luo, Yi-Hsuan Chuang, Jung-Yu Lee, Jinn-Moon Yang

**Affiliations:** 1 Institute of Bioinformatics and Systems Biology, National Yang Ming Chiao Tung University, Hsinchu, 300193 Taiwan; 2 Department of Biological Science and Technology, National Yang Ming Chiao Tung University, Hsinchu, 300193 Taiwan; 3 Center for Intelligent Drug Systems and Smart Bio-Devices, National Yang Ming Chiao Tung University, Hsinchu, 300193 Taiwan; 4 Present Address: Graphen Inc., New York, NY 10110 USA

**Keywords:** Tissue-specific gene signature, Prognostic gene signature, Omics data

## Abstract

**Background:**

The gene signatures have been considered as a promising early diagnosis and prognostic analysis to identify disease subtypes and to determine subsequent treatments. Tissue-specific gene signatures of a specific disease are an emergency requirement for precision medicine to improve the accuracy and reduce the side effects. Currently, many approaches have been proposed for identifying gene signatures for diagnosis and prognostic. However, they often lack of tissue-specific gene signatures.

**Results:**

Here, we propose a new method, consensus mutual information (CoMI) for analyzing omics data and discovering gene signatures. CoMI can identify differentially expressed genes in multiple cancer omics data for reflecting both cancer-related and tissue-specific signatures, such as *Cell growth and death* in multiple cancers, *Xenobiotics biodegradation and metabolism* in LIHC, and *Nervous system* in GBM. Our method identified 50-gene signatures effectively distinguishing the GBM patients into high- and low-risk groups (log-rank *p* = 0.006) for diagnosis and prognosis.

**Conclusions:**

Our results demonstrate that CoMI can identify significant and consistent gene signatures with tissue-specific properties and can predict clinical outcomes for interested diseases. We believe that CoMI is useful for analyzing omics data and discovering gene signatures of diseases.

**Supplementary Information:**

The online version contains supplementary material available at 10.1186/s12859-022-04682-2.

## Background

Genome-wide gene expression profiling has been used to identify genetic signatures that could be associated with the outcome of cancer patients [1]. Several different gene signatures have been developed, and many of these approaches have been shown to better define the prognosis of cancer patients as compared with conventional clinical and pathological characteristics of the tumors [1]. Some studies began with genome-wide gene expression profiling from microarray datasets or next-generation sequencing (NGS). For example, the PAM50 gene signature can use to classify breast tumors into one of these four subtypes and to predict clinical outcomes [2, 3]. Moreover, the gene signature identified from specific tissues is a promising avenue to maximize efficacy in target tissues while minimizing the safety risks of affecting unrelated tissues [4].

Many methods have been proposed to identify the gene signatures between normal and disease states [5]. The simple and common way is to use the T-test, which is a statistics-based method. According to the distributions between two states, the T-test evaluates the probability (*p*-value) in each gene. The significant difference of gene expression is often defined if the probability is less than 0.05 or 0.01. Another method is calculating the fold change (FC) of gene expression, which is not a statistical test and has no associated values for indicating the level of confidence in the genes as differentially expressed or not [6]. Therefore, some methods were developed, such as Significance Analysis of Microarrays (SAM) and Cancer Outlier Profile Analysis (COPA), to choose the biomarkers in high confidence [7–9]. The SAM approach proposed false discovery rate (FDR) to reduce genes showing significantly different expression by chance (i.e., false positive) [7, 10]. The COPA, based on the hypothesis of tumor heterogeneity, identified candidate genes overexpressing in subsets of samples by using median and median absolute deviation of gene expression profiles [8]. In another study, Parker et al. determined 50 genes (i.e., PAM50) for classified four breast subtypes with clinical means by using Predictive Analysis of Microarray (PAM) algorithm [2, 11], but this approach relied on sample labels that was difficult to propose new subtype of diseases. Recently, Gentles et al. applied CIBERSORT computational methods and PRECOG tools to identify cancer prognostic biomarkers and therapeutic targets [12], however, they only focused on the genes that related to clinical outcomes, but the ones might not be suitable for diagnosis due to the non-significant gene expression changes between the sample subgroups [13].

In this study, we hypothesize that the biological processes of tissues/organs are dysregulated during tumorigenesis, and the perturbed genes (i.e., tissue-specific genes) are often involved in corresponding functions of tissues [14–16]. To address these issues, we propose consensus mutual information (CoMI) to analyze omics data and to identify gene signatures. We utilized mutual information (MI) and gene expression distance as the basis to find the significantly and consistently expressed genes and gene signatures between normal and disease states. For multiple cancer omics data, our identified gene signatures could reflect cancer-related signatures and have tissue-specific properties (mean odds ratio = 2.89). Based on our previously developed global omics data analysis method [17], our CoMI identified gene signatures not only involved in common cancer-related progress, such as *Cell growth and death*, but also reflect tissue unique functions of *Xenobiotics biodegradation and metabolism* in LIHC and *Nervous system* in GBM. For clinical prognosis, a 50-gene signature identified by CoMI could distinguish the GBM patients into high- and low-risk groups based on gene expression patterns, and could predict clinical outcomes at 12-month survival (log-rank *p* = 0.006). We believe that our method and results are useful for analyzing omics data, discovering gene signatures with tissue-specific properties, and predicting clinical outcomes of diseases.

## Results

### CoMI for identifying gene signatures in multiple cancers

**Fig. 1:**
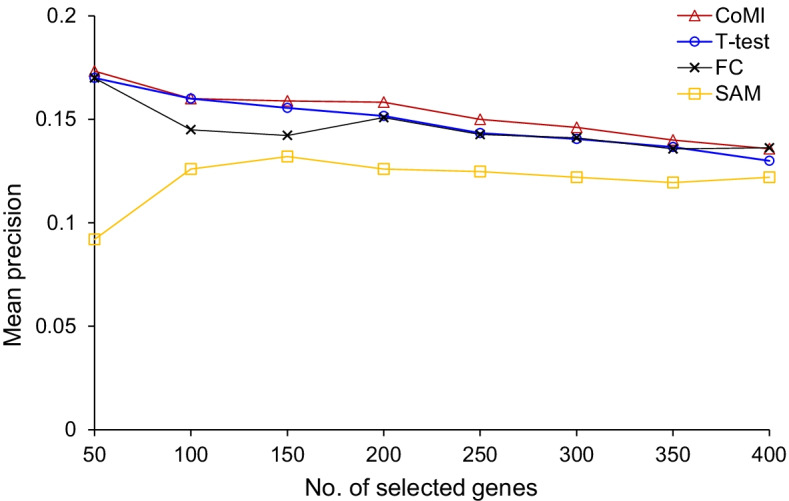
The prediction accuracies between CoMI, T-test, FC, and SAM in cancer-related genes. The mean precision of cancer-related genes recorded in HPA was selected by CoMI (red line), T-test (blue line), FC (black line), and SAM (yellow line). The predicting precision was the average of genes selected from different cut-offs in LIHC, GBM, BLCA, BRCA, and COAD

To identify consistent patterns of gene expression for gene signatures development in multiple cancers, we utilized CoMI to analyze genome-wide gene expression profiles in LIHC, GBM, BLCA, BRCA, and COAD. To evaluate the cancer associations of selected genes from CoMI in multiple cancer datasets, we collected 1675 cancer-related genes derived from HPA database [18]. The results show that the gene signatures selected from the different ranking cut-off of CoMI have a high probability to be cancer-related genes compared with T-test, FC, and Significance Analysis of Microarrays (SAM) (Fig. 1). For example, the top-ranked 200 significant genes were 190, 182, 181, and 126 genes for CoMI, T-test, FC, and SAM, respectively.

To investigate the statistical meanings of CoMI, we calculated Spearman's correlation coefficient (*ρ*) between CoMI scores and FC values, SAM scores and *p*-values of T-test on all of 20,531 genes in NGS profiles of TCGA, respectively (Additional file 1: Fig. S1). For these methods, the average *ρ* values of these five cancer types were 0.81 (FC), 0.95 (T-test), and 0.94 (SAM). These results suggest that CoMI has statistical meanings and is able to identify significantly and consistently expressed genes and disease-related gene signatures from omics data.

### Tissue-specific properties of gene signatures

**Fig. 2:**
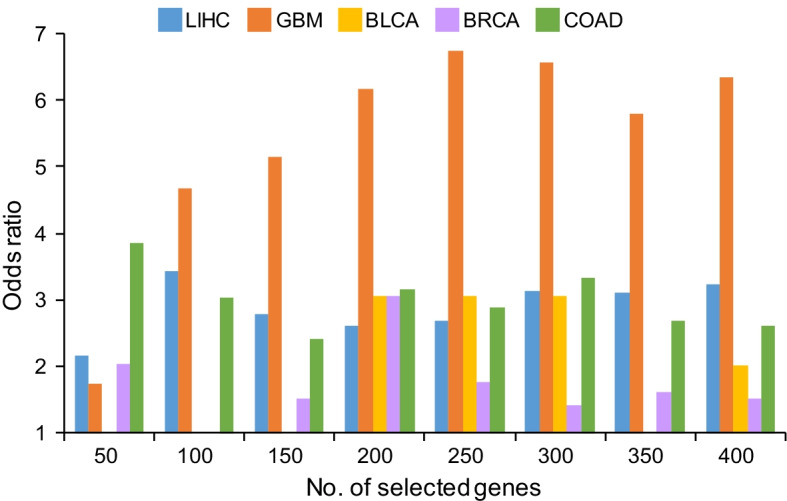
The odds ratios of tissue-specific genes selected by CoMI and T-test in different cancer types. The results of tissue-specific genes identified by CoMI and T-test in five cancer types, including LIHC (blue), GBM (orange), BLCA (yellow), BRCA (purple), and COAD (green)

To investigate the biological meanings of gene signatures identified by CoMI in different cancers, we collected tissue-specific genes from HPA with protein annotation of tissue specificity in liver, brain, urinary bladder, breast, or colon. Here, we used the odds ratio to assess whether gene signatures have tissue-specific properties or not in five cancers (Fig. 2 and Additional file 1: Fig. S2). In the comparison of CoMI and T-test, the average odds ratios of gene signatures selected from eight kinds of top-ranked thresholds were 2.89, 5.40, 1.90, 1.74, and 2.99 in LIHC, GBM, BLCA, BRCA, and COAD, respectively. For example, the top-ranked 200 genes selected by CoMI and T-test in GBM were 89 and 23 with annotated brain specificity, respectively. The odds were 0.8 (89/111) and 0.13 (23/177), then, the odds ratio was calculated as 6.15 (0.8/0.13). The results were similar when compared to SAM (Additional file 1: Fig. S2). Besides, we observed that CoMI has a lower ranking on average of descending order than the ones of T-test and SAM in all of the tissue-specific genes in each cancer (Additional file 1: Figs. S3 and S4). These results demonstrate that gene signatures identified by CoMI are more like to have tissue-specific properties.

**Fig. 3:**
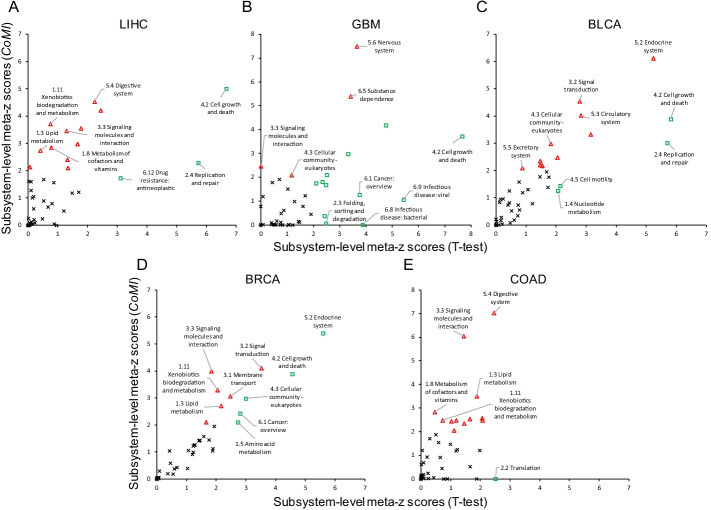
The genes identified from different cancers reflected tissue-specific pathways. The subsystem-level meta-z scores of the top-ranked 200 genes identified by CoMI and T-test in **A** LIHC, **B** GBM, **C** BLCA, **D** BRCA, and **E** COAD. The red triangle was denoted if meta-z scores > 2 and CoMI > T-test, the green square was represented if meta-z scores > 2 and T-test > CoMI

Furthermore, we used our previously developed global omics data analysis method, Hierarchical System Biology Model (HiSBiM), to investigate the involved pathways as well as biological subsystems and systems of gene signatures [17]. We observed that biological subsystems of the 200-gene signatures identified by CoMI and T-test were involved in common cancer-related pathways, such as *Cell growth and death* and *Replication and repair* in five cancer types (Fig. 3). In particular, the gene signature of LIHC identified by CoMI was enriched in *Xenobiotics biodegradation and metabolism* (meta-z score = 3.72) and *Lipid metabolism* (meta-z score = 2.74), which could reflect unique functions to liver tissue. In GBM, we observed that neurotransmission-related functions were highly enriched, such as *Nervous system* (meta-z score = 7.49) and *Signaling molecules and interaction* (meta-z score = 2.46). In addition, the *Digestive system* (meta-z score = 7.03) was highly regulated in COAD.

We also evaluated the tissue-specific properties of CoMI and COPA [9] based on HPA database and HiSBiM analysis. The results show that the genes identified by CoMI are related to both common cancer-related pathways and tissue-specific properties, such as *Endocrine system* (meta-z score = 5.40) in BRCA (Additional file 1: Fig. S5). Additionally, CoMI outperformed COPA and our results indicated that CoMI not only can identify tissue-specific gene signatures in different cancers, but also can reflect corresponding biological pathways and functions unique to those tissues.

### Clinical prognosis of gene signatures

**Fig. 4:**
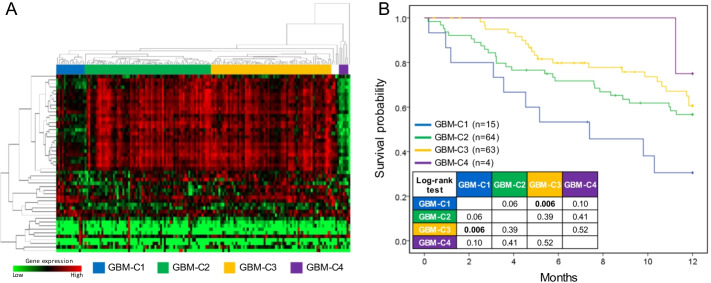
Glioblastoma multiforme prognostic gene signature. **A** The hierarchal clustering of a 50-gene signature identified by CoMI in glioblastoma multiforme (GBM) tumor samples. Based on the gene expression patterns, the expression profile could be divided into four groups, including C1 (blue), C2 (green), C3 (orange), and C4 (purple). **B** The 12-month overall survival analysis in four groups of GBM patients. The inner table showed *p*-value of log-rank test compared between four groups of patients.

The grade IV astrocytomas (i.e., GBM) are an aggressive class of brain cancer, it is difficult to treat and has a poor median 12-month overall survival, and the urgent need to develop a prognostic gene signature [19]. We selected a 50-gene signature with significant and consistent expressions by using our CoMI from the gene expression profile in GBM, as well as, five of those genes were brain tissue specificity (Table 1). According to the gene expression patterns, we found that these 50 genes could cluster 156 tumor samples into four groups (Fig. 4A). According to the 12-month overall survival analysis, the patients in GBM-C1 group (n = 15) have a significantly lower survival probability (30%) than GBM-C3 group (62%; log-rank *p* = 0.006; Fig. 4B). Moreover, we found that 70% of CoMI top-ranked 10 genes can distinguish patients into high- and low-risk groups (log-rank *p* < 0.01), such as PBK (log-rank *p* = 0.0058) and CCNB2 (log-rank *p* = 0.009), however, only five of 10 genes with log-rank *p* < 0.01 in T-test (Additional file 1: Fig. S6). These results indicated that our identified 50-gene signature could predict clinical outcomes and provide the available clues for developing the new therapeutic strategies in GBM.

**Table 1:** The 50-gene signature identified by CoMI in GBM

Genes	CoMI	*p*-value^a^	Log2 (FC)^b^	Tissue specificity^c^	GO BP	GO CC
RRM2	2.80	9.23E−33	7.48	–	Deoxyribonucleotide biosynthetic process [GO:0009263]; DNA replication [GO:0006260]; negative regulation of G0 to G1 transition [GO:0070317]	Cytosol [GO:0005829]; ribonucleoside-diphosphate reductase complex [GO:0005971]
UBE2C	2.71	4.17E−29	7.69	–	Anaphase-promoting complex-dependent catabolic process [GO:0031145]; cell division [GO:0051301]; exit from mitosis [GO:0010458]	Anaphase-promoting complex [GO:0005680]; cytosol [GO:0005829]; nucleoplasm [GO:0005654]
PBK	2.50	2.07E−27	7.30	–	Cellular response to UV [GO:0034644]; mitotic cell cycle [GO:0000278]; negative regulation of inflammatory response [GO:0050728]	Nucleus [GO:0005634]
CCNB2	2.38	5.82E−27	6.89	–	Cell division [GO:0051301]; G2/M transition of mitotic cell cycle [GO:0000086]; in utero embryonic development [GO:0001701]	Centrosome [GO:0005813]; cyclin-dependent protein kinase holoenzyme complex [GO:0000307]; cytoplasm [GO:0005737]
KIF20A	2.38	5.82E−27	6.72	–	Microtubule-based movement [GO:0007018]; microtubule bundle formation [GO:0001578]; midbody abscission [GO:0061952]	Cleavage furrow [GO:0032154]; Golgi apparatus [GO:0005794]; intercellular bridge [GO:0045171]
MYBL2	2.30	5.50E−25	7.77	–	Cellular response to leukemia inhibitory factor [GO:1990830]; mitotic cell cycle [GO:0000278]; mitotic spindle assembly [GO:0090307]	Myb complex [GO:0031523]; nucleoplasm [GO:0005654]; nucleus [GO:0005634]
NDC80	2.29	1.11E−27	6.70	–	Attachment of mitotic spindle microtubules to kinetochore [GO:0051315]; attachment of spindle microtubules to kinetochore [GO:0008608]; cell division [GO:0051301]	Centrosome [GO:0005813]; chromosome, centromeric region [GO:0000775]; condensed chromosome kinetochore [GO:0000777]
TOP2A	2.28	7.18E−26	7.60	–	Apoptotic chromosome condensation [GO:0030263]; cellular response to DNA damage stimulus [GO:0006974]; chromosome segregation [GO:0007059]	Chromosome, centromeric region [GO:0000775]; condensed chromosome [GO:0000793]; cytoplasm [GO:0005737]
DLGAP5	2.20	9.00E−25	6.93	–	Mitotic chromosome movement towards spindle pole [GO:0007079]; positive regulation of mitotic metaphase/anaphase transition [GO:0045842]; positive regulation of transcription of Notch receptor target [GO:0007221]	Centriolar satellite [GO:0034451]; cytosol [GO:0005829]; mitochondrion [GO:0005739]
BIRC5	2.12	6.81E−24	6.88	–	Cell division [GO:0051301]; chromosome segregation [GO:0007059]; cytokine-mediated signaling pathway [GO:0019221]	Centriole [GO:0005814]; chromosome, centromeric region [GO:0000775]; chromosome passenger complex [GO:0032133]
NCAPG	2.11	4.91E−28	6.53	–	Cell division [GO:0051301]; mitotic chromosome condensation [GO:0007076]	Condensed chromosome [GO:0000793]; condensed chromosome, centromeric region [GO:0000779]; condensin complex [GO:0000796]
CDC45	1.99	9.54E−21	6.64	–	DNA replication [GO:0006260]; DNA replication checkpoint [GO:0000076]; DNA replication initiation [GO:0006270]	Centrosome [GO:0005813]; ciliary basal body [GO:0036064]; cytoplasm [GO:0005737]
AURKB	1.97	1.44E−23	6.66	–	Abscission [GO:0009838]; aging [GO:0007568]; anaphase-promoting complex-dependent catabolic process [GO:0031145]	Chromocenter [GO:0010369]; chromosome passenger complex [GO:0032133]; condensed chromosome, centromeric region [GO:0000779]
MELK	1.85	2.59E−23	6.80	–	Apoptotic process [GO:0006915]; cell population proliferation [GO:0008283]; G2/M transition of mitotic cell cycle [GO:0000086]	Cell cortex [GO:0005938]; cytoplasm [GO:0005737]; membrane [GO:0016020]
TROAP	1.83	1.20E−21	6.27	–	Cell adhesion [GO:0007155]	Cytoplasm [GO:0005737]
BUB1	1.72	1.92E−26	6.07	–	Apoptotic process [GO:0006915]; cell division [GO:0051301]; cell population proliferation [GO:0008283]	Condensed chromosome kinetochore [GO:0000777]; condensed nuclear chromosome kinetochore [GO:0000778]; condensed nuclear chromosome outer kinetochore [GO:0000942]
EPR1	1.72	3.69E−22	6.77	–	Cell surface receptor signaling pathway [GO:0007166]	Integral component of membrane [GO:0016021]
HJURP	1.71	3.55E−24	6.09	–	Cell cycle [GO:0007049]; CENP-A containing nucleosome assembly [GO:0034080]; chromosome segregation [GO:0007059]	Chromosome, centromeric region [GO:0000775]; condensed chromosome kinetochore [GO:0000777]; mitochondrion [GO:0005739]
FAM64A	1.67	9.65E−21	6.72	–	Cell cycle [GO:0007049]; cell division [GO:0051301]	Nucleolus [GO:0005730]; nucleoplasm [GO:0005654]
KIF4A	1.64	9.11E−24	5.63	–	Anterograde axonal transport [GO:0008089]; antigen processing and presentation of exogenous peptide antigen via MHC class II [GO:0019886]; microtubule-based movement [GO:0007018]	Axon cytoplasm [GO:1904115]; chromosome [GO:0005694]; cytoplasm [GO:0005737]
ASF1B	1.63	1.00E−24	5.80	–	Blastocyst hatching [GO:0001835]; cell differentiation [GO:0030154]; DNA replication-dependent nucleosome assembly [GO:0006335]	Nuclear chromatin [GO:0000790]; nucleoplasm [GO:0005654]; protein-containing complex [GO:0032991]
NUSAP1	1.54	8.64E−24	5.63	–	Establishment of mitotic spindle localization [GO:0040001]; mitotic chromosome condensation [GO:0007076]; mitotic cytokinesis [GO:0000281]	Chromosome [GO:0005694]; cytoplasm [GO:0005737]; microtubule [GO:0005874]
CEP55	1.53	1.10E−26	5.42	–	Cranial skeletal system development [GO:1904888]; establishment of protein localization [GO:0045184]; midbody abscission [GO:0061952]	Centriolar satellite [GO:0034451]; centriole [GO:0005814]; centrosome [GO:0005813]
CENPA	1.52	5.29E−22	5.55	–	CENP-A containing nucleosome assembly [GO:0034080]; establishment of mitotic spindle orientation [GO:0000132]; kinetochore assembly [GO:0051382]	Chromosome, centromeric region [GO:0000775]; condensed chromosome inner kinetochore [GO:0000939]; condensed nuclear chromosome, centromeric region [GO:0000780]
SHOX2	1.48	6.10E−16	6.91	–	Cardiac atrium morphogenesis [GO:0003209]; cartilage development involved in endochondral bone morphogenesis [GO:0060351]; chondrocyte development [GO:0002063]	Nuclear chromatin [GO:0000790]
KIFC1	1.44	3.38E−19	5.72	–	Cell division [GO:0051301]; microtubule-based movement [GO:0007018]; mitotic metaphase plate congression [GO:0007080]	Early endosome [GO:0005769]; kinesin complex [GO:0005871]; membrane [GO:0016020]
KIAA0101	1.43	8.07E−21	5.95	–	Cellular response to DNA damage stimulus [GO:0006974]; centrosome cycle [GO:0007098]; DNA replication [GO:0006260]	Centrosome [GO:0005813]; nucleoplasm [GO:0005654]; nucleus [GO:0005634]
KIF2C	1.33	3.31E−20	5.29	–	Antigen processing and presentation of exogenous peptide antigen via MHC class II [GO:0019886]; attachment of mitotic spindle microtubules to kinetochore [GO:0051315]; cell division [GO:0051301]	Centrosome [GO:0005813]; chromosome, centromeric region [GO:0000775]; condensed chromosome kinetochore [GO:0000777]
ATP8A2	1.31	8.43E−13	6.85	Brain	Aging [GO:0007568]; axonogenesis [GO:0007409]; detection of light stimulus involved in visual perception [GO:0050908]	Endosome [GO:0005768]; Golgi apparatus [GO:0005794]; integral component of membrane [GO:0016021]
CENPK	1.31	4.27E−21	5.26	–	CENP-A containing nucleosome assembly [GO:0034080]; kinetochore assembly [GO:0051382]; mitotic sister chromatid segregation [GO:0000070]	Condensed nuclear chromosome inner kinetochore [GO:0000941]; cytosol [GO:0005829]; nucleoplasm [GO:0005654]
FOXM1	1.26	7.18E−19	4.95	–	DNA damage response, signal transduction by p53 class mediator resulting in transcription of p21 class mediator [GO:0006978]; DNA repair [GO:0006281]; G2/M transition of mitotic cell cycle [GO:0000086]	Nuclear chromatin [GO:0000790]; nucleoplasm [GO:0005654]; nucleus [GO:0005634]
GTSE1	1.23	7.44E−19	5.23	–	DNA damage response, signal transduction by p53 class mediator resulting in cell cycle arrest [GO:0006977]; microtubule-based process [GO:0007017]; positive regulation of cell migration [GO:0030335]	Cytoplasmic microtubule [GO:0005881]; cytosol [GO:0005829]; membrane [GO:0016020]
IGFBP2	1.22	1.01E−14	5.58	–	Aging [GO:0007568]; cellular protein metabolic process [GO:0044267]; cellular response to hormone stimulus [GO:0032870]	Apical plasma membrane [GO:0016324]; cytoplasmic vesicle [GO:0031410]; extracellular exosome [GO:0070062]
KIF14	1.22	7.45E−18	5.20	–	Activation of protein kinase activity [GO:0032147]; cell division [GO:0051301]; cell proliferation in forebrain [GO:0021846]	Cytosol [GO:0005829]; Flemming body [GO:0090543]; kinesin complex [GO:0005871]
MKI67	1.22	1.18E−16	5.67	–	Cell cycle [GO:0007049]; cell population proliferation [GO:0008283]; regulation of chromatin organization [GO:1902275]	Chromosome [GO:0005694]; membrane [GO:0016020]; nuclear body [GO:0016604]
F2R	1.21	9.11E−24	4.72	–	Activation of cysteine-type endopeptidase activity involved in apoptotic process [GO:0006919]; activation of MAPKK activity [GO:0000186]; anatomical structure morphogenesis [GO:0009653]	Caveola [GO:0005901]; cell surface [GO:0009986]; early endosome [GO:0005769]
FAM111B	1.19	1.50E−17	5.47	–	DNA replication [GO:0006260]	Chromatin [GO:0000785]; nucleus [GO:0005634]
PNMA5	1.18	2.62E−14	6.55	Brain	Positive regulation of apoptotic process [GO:0043065]	–
PRKCG	1.18	1.06E−11	7.41	Brain	Chemical synaptic transmission [GO:0007268]; chemosensory behavior [GO:0007635]; innervation [GO:0060384]	Calyx of Held [GO:0044305]; cell–cell junction [GO:0005911]; cytosol [GO:0005829]
MLF1IP	1.17	2.29E−19	4.91	–	CENP-A containing nucleosome assembly [GO:0034080]; chordate embryonic development [GO:0043009]; viral process [GO:0016032]	Centriolar satellite [GO:0034451]; condensed chromosome kinetochore [GO:0000777]; cytosol [GO:0005829]
CDCA2	1.17	5.25E−18	5.29	–	Cell cycle [GO:0007049]; cell division [GO:0051301]; chromosome segregation [GO:0007059]	Chromosome [GO:0005694]; cytosol [GO:0005829]; nucleoplasm [GO:0005654]
E2F8	1.16	7.40E−19	5.30	–	Cell cycle comprising mitosis without cytokinesis [GO:0033301]; cell population proliferation [GO:0008283]; chorionic trophoblast cell differentiation [GO:0060718]	Cytosol [GO:0005829]; nuclear chromatin [GO:0000790]; nucleolus [GO:0005730]
EZH2	1.15	2.37E−21	4.80	–	Cardiac muscle hypertrophy in response to stress [GO:0014898]; cellular response to hydrogen peroxide [GO:0070301]; cellular response to trichostatin A [GO:0035984]	Chromosome, telomeric region [GO:0000781]; cytoplasm [GO:0005737]; ESC/E(Z) complex [GO:0035098]
C1QL3	1.14	1.54E−11	6.92	Brain	Regulation of synapse organization [GO:0050807]	Collagen trimer [GO:0005581]; extracellular region [GO:0005576]
CDK1	1.14	2.71E−19	4.91	–	Activation of MAPK activity [GO:0000187]; anaphase-promoting complex-dependent catabolic process [GO:0031145]; animal organ regeneration [GO:0031100]	Centrosome [GO:0005813]; cyclin B1-CDK1 complex [GO:0097125]; cyclin-dependent protein kinase holoenzyme complex [GO:0000307]
BUB1B	1.13	9.46E−19	5.12	–	Anaphase-promoting complex-dependent catabolic process [GO:0031145]; apoptotic process [GO:0006915]; cell division [GO:0051301]	Anaphase-promoting complex [GO:0005680]; condensed chromosome kinetochore [GO:0000777]; condensed chromosome outer kinetochore [GO:0000940]
KIF18A	1.13	1.24E−18	4.90	–	Antigen processing and presentation of exogenous peptide antigen via MHC class II [GO:0019886]; cellular response to estradiol stimulus [GO:0071392]; male meiotic nuclear division [GO:0007140]	Caveola [GO:0005901]; cytoplasm [GO:0005737]; cytosol [GO:0005829]
SST	1.12	3.64E−09	6.66	Brain	Cell–cell signaling [GO:0007267]; cell surface receptor signaling pathway [GO:0007166]; chemical synaptic transmission [GO:0007268]	Extracellular region [GO:0005576]; extracellular space [GO:0005615]; neuronal cell body [GO:0043025]
E2F2	1.11	2.48E−14	5.09	–	Cell cycle [GO:0007049]; intrinsic apoptotic signaling pathway by p53 class mediator [GO:0072332]; lens fiber cell apoptotic process [GO:1990086]	Nuclear chromatin [GO:0000790]; nucleoplasm [GO:0005654]; nucleus [GO:0005634]
RYR2	1.11	6.41E−12	7.23	–	Calcium ion transport [GO:0006816]; calcium ion transport into cytosol [GO:0060402]; calcium-mediated signaling [GO:0019722]	Calcium channel complex [GO:0034704]; cytoplasmic vesicle membrane [GO:0030659]; junctional sarcoplasmic reticulum membrane [GO:0014701]

^a^Calculated by T-test

^b^Absolute values of log2(fold change)

^c^RNA tissue specificity recorded in HPA database

## Discussion

Genome-wide gene expression profiling has been used to identify genetic signatures that could be associated with the outcome of cancer patients [1]. Some studies using genome-wide gene expression profiling for developing gene signatures, and many of these approaches have shown to better define the prognosis of cancer patients, such as the PAM50 gene signature can use to classify breast tumors into four subtypes and to predict clinical prognosis [2, 3].

In this paper, we propose consensus mutual information (CoMI) to analyze omics data and to identify gene signatures. For multiple cancer omics data, we used CoMI to identify gene signatures, and those genes could reflect cancer-related signatures and have tissue-specific properties. In general, normal cells perform the function they are meant to perform, whereas cancer cells may not execute these functions. For example, cancerous thyroid cells may not produce thyroid hormone [20], and cancerous white blood cells are not functioning as they should [21]. On the other hand, the genes recorded in cancer hallmarks are often activated in cancer cells [22].

Here, we hypothesize that tissue-specific genes are often related to lost-of-tissue functions which are often down-regulated in the disease states. Our CoMI can identify these tissue-specific genes which are significant change (*S*_*Dist*_ score) and have consensus expressed values (*S*_*MI*_) within cancer and normal samples. The changes between cancer and normal samples were quantified by considering global gene expression values for all genes in omics data. Based on our scoring function, CoMI identified the liver-specific genes, such as CYP1A2 (CoMI score = 0.99 and FC = − 7.76), CYP2C8 (CoMI score = 0.71 and FC = − 4.14), and CYP3A4 (CoMI score = 0.55 and FC = − 5.85), are the members of the cytochrome P450 family involving in Lipid metabolism (meta-z score = 2.74) as well as Xenobiotics biodegradation and metabolism (meta-z score = 3.72) in LIHC. Additionally, CoMI also identified gene SLC22A1 (CoMI score = 0.53 and FC = − 4.81) involving in liver unique functions, that is, bile secretion of Digestive system (meta-z score = 4.54). Conversely, MAP2K1, an essential component of the MAP kinase signal transduction pathway related to cancer hallmark, was not a tissue-specific gene. We found that MAP2K1 significantly changed in most cancers, but its expression values are not consistent (CoMI score = 0.20). These results show that our CoMI identified gene signatures not only involved in cancer-related progress, such as *Cell growth and death*, but also reflect tissue unique functions of *Nervous system* (meta-z score = 7.49) in GBM. For clinical prognosis, our identified 50-gene signature could stratify GBM patients into high- and low-risk groups, and could predict clinical outcomes with 12-month survival.

There were some limitations to the current study. Firstly, the omics data (i.e., NGS) is only collected from TCGA, and different sources and platforms, such as microarray, are needed to use to validate our method and identified gene signatures. Secondly, the results of this study are mostly based on bioinformatics analysis and predictions, and further experiments are needed to prove the tissue-specific properties of our identified gene signatures. Thirdly, although we identified prognostic gene signatures, those genes remain to be further explored in our future work.

## Conclusion

In summary, we have proposed CoMI for analyzing omics data and discovering gene signatures. Our method accomplished the identification of genes and gene signatures, which have consistently and significantly changed between normal and disease states. Our results indicated that CoMI could identify gene signatures with tissue-specific properties for interested diseases, and is able to be applied to predict clinical prognosis.

## Methods

**Fig. 5:**
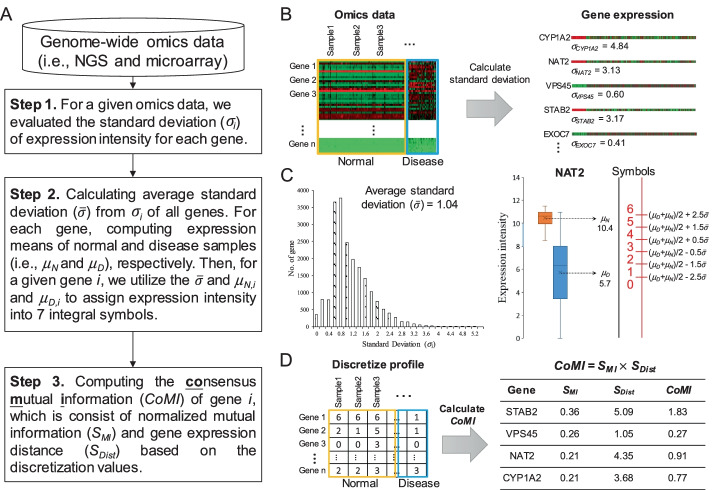
Overview of consensus mutual information. **A** Flowchart describing the main procedure. **B** We firstly evaluated the standard deviation ($\sigma_{i}$) of gene expression intensity for each gene in a given omics data. **C** We then computing the average standard deviation ($\overline{\sigma }$) from $\sigma_{i}$ of all genes, as well as expression means of normal and disease samples (i.e., *μ*_*N*_ and *μ*_*D*_), respectively, in each gene. For a given gene *i* in sample *j*, we assigned expression intensity into 7 integral symbols by considering the $\overline{\sigma }$ and its *μ*_*N*_ and *μ*_*D*._ **D** The gene expression values were converted to discretized integer symbol ranging from 0 to 6. The highly expressed genes were assigned to the highest symbol 6 and lowly expressed genes were assigned to the lowest symbol 0. (D) The calculation of consensus mutual information (CoMI) values of all genes

To identify significantly and consistently expressed genes and gene signatures, we proposed a method, consensus mutual information (called CoMI), for analyzing omics data between normal and disease samples (Fig. 5A). We first calculated gene expression variations for each gene in a given omics data (Fig. 5B). For the evaluating of the consensus gene expression in normal and tissue states, we transferred the continuous gene expression to discrete integer symbols based on expression variations and intensities (Fig. 5C). Finally, we computed CoMI score for all genes, which can be used as a measure for significantly and consistently expressed genes and gene signatures between two states (Fig. 5D).

### Omics and validation data

Here, we collected omics data (i.e. NGS) in LIHC, GBM, BLCA, BRCA, and COAD from The Cancer Genome Atlas (TCGA) databases [23]. These datasets contain 228 normal and 2315 tumor samples. To evaluate our method and compare to T-test and FC method, we collected the 1675 cancer-related genes derived from the Human Protein Atlas (HPA) database [18]. The tissue-specific genes were collected from HPA with protein annotation of tissue specificity in liver (409 genes), brain (1313 genes), urinary bladder (56 genes), breast (82 genes), or colon (147 genes). Finally, we calculated the *p*-values (i.e., T-test), fold changes, and SAM scores of all genes by using limma and samr R package [24, 25].

### Discretization

To consider the readily quantifiable and significant expressions in the disease state of genes and gene signatures, we firstly evaluated the standard deviation ($\sigma_{i}$) of expression intensity of the gene *i* (Fig. 5B). Then, we calculated the average standard deviation ($\overline{\sigma }$) from $\sigma_{i}$ of all genes (Fig. 5C). For each gene, computing expression means of normal (*μ*_*N*_) and disease (*μ*_*D*_) samples, respectively. For a given gene *i* in sample *j*, we utilize the average standard deviation ($\overline{\sigma }$), average expression (*μ*_*N,i*_ and *μ*_*D,i*_) to assign expression intensity (*EI*_*i,j*_) into an integral symbol (*ES*_*i,j*_) by using the following equations:

1 $$ES_{i,j} = \left\{ \begin{aligned} & 6, \;\;if EI_{i,j} > \frac{{\mu_{D,i} +\mu_{N,i} }}{2} + 2.5\overline{\sigma } \\ & 5, \;\;if \frac{{\mu_{D,i} +\mu_{N,i} }}{2} + 1.5\overline{\sigma } < EI_{i,j} \le \frac{{\mu_{D,i} +\mu_{N,i} }}{2} + 2.5\overline{\sigma } \\ & 4, \;\;if \frac{{\mu_{D,i} +\mu_{N,i} }}{2} + 0.5\overline{\sigma } < EI_{i,j} \le \frac{{\mu_{D,i} +\mu_{N,i} }}{2} + 1.5\overline{\sigma } \\ & 3, \;\;if \frac{{\mu_{D,i} +\mu_{N,i} }}{2} - 0.5\overline{\sigma } < EI_{i,j} \le \frac{{\mu_{D,i} +\mu_{N,i} }}{2} + 0.5\overline{\sigma } \\ & 2, \;\;if \frac{{\mu_{D,i} +\mu_{N,i} }}{2} - 1.5\overline{\sigma } < EI_{i,j} \le \frac{{\mu_{D,i} +\mu_{N,i} }}{2} - 0.5\overline{\sigma } \\ & 1, \;\;if \frac{{\mu_{D,i} +\mu_{N,i} }}{2} - 2.5\overline{\sigma } < EI_{i,j} \le \frac{{\mu_{D,i} +\mu_{N,i} }}{2} - 1.5\overline{\sigma } \\ & 0,\;\; if EI_{i,j} \le \frac{{\mu_{D,i} +\mu_{N,i} }}{2} - 2.5\overline{\sigma } \\ \end{aligned} \right.$$

The gene expression values were converted to discretized integer symbols ranging from 0 to 6. The highly expressed gene was assigned to the symbol 6 and lowly expressed gene was assigned to the symbol 0. For instance, the $\overline{\sigma }$ is 1.04 in LIHC, and the *μ*_*N*_ and *μ*_*D*_ of the gene NAT2 are 10.4 and 5.7, respectively. The expression intensity (*EI*) of NAT2 is 10.8 in sample TCGA-DD-AAE3-01A-11R-A41C-07, which satisfies the $EI_{i,j} > \frac{{\mu_{D,i} +\mu_{N,i} }}{2} + 2.5\overline{\sigma }$ (i.e., 10.7), therefore, we assign the *ES*_*NAT2,TCGA-DD-AAE3-01A-11R-A41C-07*_ value to 6.

### CoMI: Consensus mutual information

For each gene *i* in a given omics data, we evaluated its consensus mutual information (CoMI) and to identify significantly and consistently expressed genes and gene signatures between normal and disease states (Fig. 5D). The CoMI of a gene is defined as:

2 $$CoMI = S_{MI} \times S_{Dist}$$

where *S*_*MI*_ is gene expression difference between two states using mutual information; *S*_*Dist*_ is the gene expression distance between two states by using mean distance. For the gene *i*, the *S*_*MI*_ is given as:

3 $$S_{MI} = \mathop \sum \limits_{y = 1}^{Y} \mathop \sum \limits_{x = 1}^{X} p\left( {x,y} \right){\text{log}}\left( {\frac{{p\left( {x,y} \right)}}{p\left( x \right)p\left( y \right)}} \right)$$

where *p*(*x,y*) is the probability of gene *i* in symbol *x* and state *y*; *p*(*x*) is the fraction of gene *i* in symbol *x*, and *p*(*y*) is the fraction of samples in state *y*. *Y* is the number of states (here, Y = 2 for normal and disease states), and *X* is the number of symbols (here, X = 7). The *S*_*Dist*_ is given as:

4 $$S_{Dist} = \left( {\frac{{\mathop \sum \nolimits_{n \in y1} ES_{i,n} }}{N} - \frac{{\mathop \sum \nolimits_{d \in y2} ES_{i,d} }}{D}} \right)$$

where *N* and *D* are numbers of samples in the normal (*y*_*1*_) and disease (*y*_*2*_) states, respectively; $ES_{i,n}$ and $ES_{i,d}$ are the integral symbols of gene *i* at the sample for normal and disease states, respectively.

According to the equation of mutual information, the *S*_*MI*_ is related to the ratio between normal and cancer samples. *S*_*MI*_ is also related to the overlap of integer symbols between normal and cancer samples. For example, *S*_*MI*_ = 1, while *p(normal)* = *p(cancer)* and there is no overlap of integer symbols between normal and cancer samples; *S*_*MI*_ is from 0.3 to 0.5, while *p(normal)* = *p(cancer)* and half of the integer symbols in normal and cancer samples are the same; *S*_*MI*_ = 1, while *p(normal)* = *p(cancer)* and there is no overlap of integer symbols between normal and cancer samples; *S*_*MI*_ = 0.44, while 10 × *p(normal)* = *p(cancer)* and there is no overlap of integer symbols between normal and cancer samples; *S*_*MI*_ is from 0.3 to 0.4, while 10 × *p(normal)* = *p(cancer)* and half of the integer symbols in normal samples are the same as integer symbols in cancer samples; *S*_*MI*_ = 0.28, while 20 × *p(normal)* = *p(cancer)* and there is no overlap of integer symbols between normal and cancer samples. In our collected NGS data in LIHC, GBM, BLCA, BRCA, and COAD from TCGA databases, there are 228 normal and 2315 tumor samples and the expected maxima of *S*_*MI*_ might be 0.44. Here, we assumed that CoMI > 0.6 might be a suitable cut-off which could ensure only half of the integer symbols in normal samples are the same as integer symbols in cancer samples and the *S*_*Dist*_ is greater than 2 (Additional file 1: Fig. S7).

Moreover, we found that both *S*_*MI*_ and *S*_*Dist*_ could be used as good indexes to identify the genes with tissue-specific properties (Additional file 1: Fig. S8). *S*_*Dist*_ focuses on the distance between two states and is more related to the tissue-specific genes in our five data sets. In this study, we provide a scoring system that has a reliable discretizing method and consider both distances between two states and mutual information to identify gene signatures. Using *S*_*MI*_ and *S*_*Dist*_ could evaluate the distance, significantly and consistently expressed value of the gene between normal and disease states under the same scale.

## Supplementary Information

**Additional file 1: Fig. S1**. The correlations of the ranking of all genes were compared between CoMI, T-test, FC, and SAM in five cancer types. **Fig. S2**. The odds ratios of tissue-specific genes selected by CoMI and SAM in five cancer types, including LIHC, GBM, BRCA, and COAD. **Fig. S3**. Boxplot for the results of the ranking of tissue-specific genes identified by CoMI and T-test in five cancer types. **Fig. S4**. Boxplot for the results of the ranking of tissue-specific genes identified by CoMI and SAM in five cancer types. **Fig. S5**. Comparisons between CoMI and COPA for tissue-specific properties in five cancer types. **Fig. S6**. Kaplan–Meier plots representing patients stratified by the auto-select best cutoff of top-ranked 10 genes identified by CoMI in GBM. **Fig. S7. **The distributions of CoMI values with different *S*_*MI*_ and *S*_*Dist*_ values. **Fig. S8. **The relationship between *S*_*MI*_, *S*_*Dist*_, and tissue-specific genes.

## Data Availability

The omics data of LIHC, GBM, BLCA, BRCA, and COAD and clinical information were collected from TCGA cohort of Genomic Data Commons Data Portal database (https://portal.gdc.cancer.gov/). Kaplan–Meier analysis was performed by using R tool which could be obtained from R Project (https://www.r-project.org/).

## Abbreviations

CoMI: Consensus mutual information
FC: Fold change
NGS: Next-generation sequencing
HPA: Human protein atlas
TCGA: The cancer genome atlas
LIHC: Liver hepatocellular carcinoma
GBM: Glioblastoma multiforme
BLCA: Bladder urothelial carcinoma
BRCA: Breast invasive carcinoma
COAD: Colon adenocarcinoma
