# Supplementary material for: CoMI: consensus mutual information for tissue-specific gene signatures
Source: BMC Bioinformatics. 2022 Apr 19;22(Suppl 10):624. doi: 10.1186/s12859-022-04682-2 (PMC9019939; doi:10.1186/s12859-022-04682-2)
Supplement: Supplementary file 1 — Additional file 1: Fig. S1. The correlations of the ranking of all genes were compared between CoMI, T-test, FC, and SAM in five cancer types. Fig. S2. The odds ratios of tissue-specific genes selected by CoMI and SAM in five cancer types, including LIHC, GBM, BRCA, and COAD. Fig. S3. Boxplot for the results of the ranking of tissue-specific genes identified by CoMI and T-test in five cancer types. Fig. S4. Boxplot for the results of the ranking of tissue-specific genes identified by CoMI and SAM in five cancer types. Fig. S5. Comparisons between CoMI and COPA for tissue-specific properties in five cancer types. Fig. S6. Kaplan–Meier plots representing patients stratified by the auto-select best cutoff of top-ranked 10 genes identified by CoMI in GBM. Fig. S7. The distributions of CoMI values with different SMI and SDist values. Fig. S8. The relationship between SMI, SDist, and tissue-specific genes. [file 12859_2022_4682_MOESM1_ESM.docx]

**CoMI: Consensus Mutual Information for Tissue-specific Gene Signatures**

**(Supplementary Material)**

Sing-Han Huang^1,4^, Yu-Shu Lo^1^, Yong-Chun Luo^1^, Yi-Hsuan Chuang^1^, Jung-Yu Lee^1^ and Jinn-Moon Yang^1,2,3*^

^1^Institute of Bioinformatics and Systems Biology, National Yang Ming Chiao Tung University, Hsinchu 300193, Taiwan.

^2^Department of Biological Science and Technology, National Yang Ming Chiao Tung University, Hsinchu 300193, Taiwan.

^3^Center for Intelligent Drug Systems and Smart Bio-devices, National Yang Ming Chiao Tung University, Hsinchu 300193, Taiwan.

^4^Present address: Graphen Inc., New York, NY 10110, USA.

*Correspondence: moon@faculty.nctu.edu.tw (J.-M.Y.)

**Table of Contents**

**I. Additional Figures**

Additional Figure S1. The correlations of the ranking of all genes were compared between CoMI, T-test, FC, and SAM in five cancer types.

Additional Figure S2. The odds ratios of tissue-specific genes selected by CoMI and SAM in five cancer types, including LIHC, GBM, BRCA, and COAD.

Additional Figure S3. Boxplot for the results of the ranking of tissue-specific genes identified by CoMI and T-test in five cancer types.

Additional Figure S4. Boxplot for the results of the ranking of tissue-specific genes identified by CoMI and SAM in five cancer types.

Additional Figure S5. Comparisons between CoMI and COPA for tissue-specific properties in five cancer types.

Additional Figure S6. Kaplan–Meier plots representing patients stratified by the auto-select best cutoff of top-ranked 10 genes identified by CoMI in GBM.

Additional Figure S7. The distributions of CoMI values with different *S_MI_* and *S_Dist_* values.

Additional Figure S8. The relationship between *S_MI_*, *S_Dist_*, and tissue-specific genes.

**I. Additional Figures**

**Additional Figure S1**. **The correlations of the ranking of all genes were compared between CoMI, T-test, FC, and SAM in five cancer types.** The spearman's *ρ* was used to evaluate gene correlations in different approaches.

**Additional Figure S2**. **The odds ratios of tissue-specific genes selected by CoMI and SAM in five cancer types**, including LIHC (blue), GBM (orange), BRCA (purple), and COAD (green). Please note that none of top-ranked 400 genes selected by SAM were bladder-specific genes in BLCA.

**Additional Figure S3.** **Boxplot for the results of the ranking of tissue-specific genes identified by CoMI and T-test in five cancer types.** The colors of different cancers were denoted in blue (LIHC), orange (GBM), yellow (BLCA), purple (BRCA), and green (COAD). Tissue-specific genes were collected from HPA with protein annotation of tissue specificity in liver, brain, urinary bladder, breast, or colon. Here, *p*-values were converted to -log10(*p*) for comparing with CoMI in descending order.

**Additional Figure S4.** **Boxplot for the results of the ranking of tissue-specific genes identified by CoMI and SAM in five cancer types.** The colors of different cancers were denoted in blue (LIHC), orange (GBM), yellow (BLCA), purple (BRCA), and green (COAD). Tissue-specific genes were collected from HPA with protein annotation of tissue specificity in liver, brain, urinary bladder, breast, and colon.

**Additional Figure S5.** **Comparisons between CoMI and COPA for tissue-specific properties in five cancer types.** The subsystem-level meta-z scores of the DEGs identified by CoMI and COPA in (A) LIHC, (B) GBM, (C) BLCA, (D) BRCA, and (E) COAD using Hierarchical System Biology Model (HiSBiM) [7]. The red triangle was denoted if meta-z scores > 2 and CoMI is better than COPA, the green square was represented if meta-z scores > 2 and COPA is better than CoMI.

**Additional Figure S6.** **Kaplan–Meier plots representing patients stratified by the auto-select best cutoff of top-ranked 10 genes identified by CoMI in GBM.**

**Additional Figure S7. The distributions of CoMI values with different *S_MI_* and *S_Dist_* values.**

**
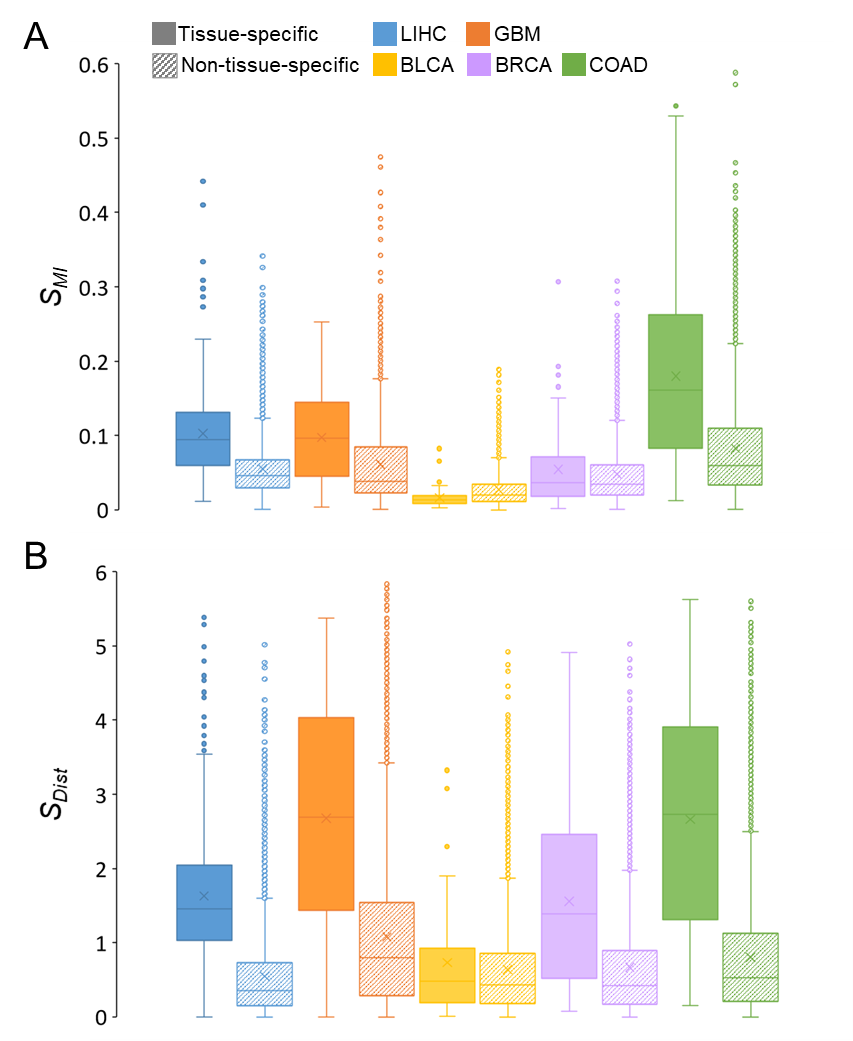
**

**Additional Figure S8. The relationship between *S_MI_* and *S_Dist_* and tissue-specific genes.**
